# Pre-clinical Models for Studying the Interaction Between Mesenchymal Stromal Cells and Cancer Cells and the Induction of Stemness

**DOI:** 10.3389/fonc.2019.00305

**Published:** 2019-04-30

**Authors:** Sofia Avnet, Silvia Lemma, Margherita Cortini, Gemma Di Pompo, Francesca Perut, Nicola Baldini

**Affiliations:** ^1^Orthopaedic Pathophysiology and Regenerative Medicine Unit, IRCCS Istituto Ortopedico Rizzoli, Bologna, Italy; ^2^Department of Biomedical and Neuromotor Sciences, University of Bologna, Bologna, Italy

**Keywords:** mesenchymal stromal cells, tumour microenvironment, stemness, secretome, 3D models, metastasis

## Abstract

Mesenchymal stromal cells (MSC) have essential functions in building and supporting the tumour microenvironment, providing metastatic niches, and maintaining cancer hallmarks, and it is increasingly evident that the study of the role of MSC in cancer is crucial for paving the way to clinical opportunities for novel anti-cancer therapies. To date, the vast majority of preclinical models that have been used for studying the effect of reactive MSC on cancer growth, metastasis, and response to therapy has been mainly based on *in vitro* flat biology, including the co-culturing with cell compartmentalization or with cell-to-cell contact, and on *in vivo* cancer models with different routes of MSC inoculation. More complex *in vitro* 3D models based on spheroid structures that are formed by intermingled MSC and tumour cells are also capturing the interest in cancer research. These are innovative culture systems tailored on the specific tumour type and that can be combined with a synthetic extracellular matrix, or included in *in silico* technologies, to more properly mimic the *in vivo* biological, spatial, biochemical, and biophysical features of tumour tissues. In this review, we summarized the most popular and currently available preclinical models for evaluating the role of MSC in cancer and their specific suitability, for example, in assaying the MSC-driven induction of epithelial-to-mesenchymal transition or of stem-like traits in cancer cells. Finally, we enlightened the need to carefully consider those parameters that might unintentionally strongly affect the secretome in MSC-cancer interplay and introduce confounding variables for the interpretation of results.

## Introduction

Cancer is a complex disease that thrives in a heterogeneous and adaptive tumour microenvironment (TME) admixed with reactive elements surrounding or infiltrating the tumour cells. Among these, endothelial, immune, and mesenchymal stromal cells (MSC) or cancer-associated fibroblasts (CAF) are frequently observed, playing an important role during carcinogenesis and cancer progression (1, 2). As a part of the tumour-supporting mesenchymal stroma, CAF have been suggested to originate from MSC, thereby sharing several features (3–5). For the distinguishing between MSC and CAF we recommend other previous review (5), since in this review, we will focus on MSC. Regardless the tissue origins, MSC are inherently tumour-homing and are a considerable component of the general host response to tissue damage caused by cancer cells. Cancer has been associated with MSC at all stages of disease progression with contradicting conclusions. Indeed, MSC have been also shown to have anti-cancer activities (6). More often, MSC are considered a foe in cancer for the immunosuppressive ability that creates a protective milieu for tumour cells by recruiting immunosuppressive tumour-associated macrophage TAM (7), for the promotion of tumour angiogenesis, proliferation, and metastasis, but also tumour dormancy and drug resistance (5).

## CSC and MSC

Under physiological conditions, MSC have a major role in the maintenance of stem cell niches, like for the hematopoietic niche (8). Similarly, in cancer, MSC are relevant for the formation and maintenance of cancer stem cells (CSC). CSC are considered quiescent cells that have been isolated from a number of tumours (e.g., hematopoietic malignancies, breast carcinoma, glioblastoma, and sarcomas) by using different techniques (9–12).

Research on CSC has defined them as the driving force in tumour formation as they are characterized by self-renewal ability and can give rise to heterogeneous lineages that recapitulate the main tumour features (13). Yet, it has gradually become clear that CSC, like normal stem cells, do not necessarily have to be rare and/or quiescent; multiple examples now show that they can be abundant and can proliferate vigorously. Furthermore, it is emerging that stem cell hierarchies may be much more plastic than previously appreciated, a phenomenon that complicates the identification and eradication of CSC (14).

Despite recent data are therefore questioning the validity of the CSC model, the CSC-MSC interaction is well documented and is crucial for 3D growth and stemness of tumour cells (15), or for the generation of an hybrid MSC-CSC cell populations, occurring via entosis, i.e., a form of cell-to-cell internalization, or via fusion, as demonstrated in different tumours (16–20). Indeed, the selection processes of hybrid cells after MSC–tumour cell fusion contribute to CSC development (20, 21). CSC-MSC interaction occurs via soluble factors (22), exosomes, or direct interaction (23). Another form of contact has been described for glioblastoma, where the CSC state is regulated by a transient interaction between cancer cells and platelets. This contact induces an epithelial-to-mesenchymal (EMT) transition program leading to the expression of mesenchymal features which may coincide with a CSC-like state. In turn, once CSC have seeded a distant organ, they can orchestrate stromal cells to their needs (23) as CSC may promote, as an example, the release of TGF-β by MSC that further fosters EMT and increases the CSC stem-like state (24).

Given their mesenchymal origin, EMT cannot be proposed in sarcomas. However, after we demonstrated the existence of CSC (25), we showed that, by using a 2D co-culture system that also included CSC spheroids, MSC increased the CSC migratory capacity via TGF-β1 that, in turn, stimulated the secretion of the pro-inflammatory cytokine IL-6 that fostered osteosarcoma stemness and aggressiveness (26). Blocking the TGF- β1 signalling pathway in the same MSC-CSC co-cultures inhibited the CSC dedifferentiation, clonogenicity, and self-renewal capacity (27).

It follows that the study of the interaction between MSC and cancer cells, with or without stem-like properties, is crucial to bring out clinical opportunities for new cancer therapies. However, the set up of the appropriate models according to the specific study aim is crucial. Here, we summarized the currently available and most popular preclinical models and their specific features for modelling MSC-cancer cells interplays.

## *In vitro* Models

The set up of pre-clinical models with MSC requires *in vitro* expansion thereby possibly causing meaningful changes in MSC behaviour, and affecting the interpretation of results. However, this is a bias that, to date, cannot be overcome to meet the demand for MSC-cancer cells preclinical modelling. The current *in vitro* preclinical models are mainly based on two-dimensional (2D) surfaces and include co-culture systems. These type of models have the enormous advantage to easily allow the control of the experimental conditions, and the analysis of expression of specific molecular signalling that can be distinguished between the two different cell populations. The first and simplest example is the treatment of cancer cells with conditioned medium of MSC cultures and is useful to study the effect of MSC-secreted soluble factors on cancer cell behaviour (28–30). A more complex system is based on the use of transwell with the two cell types seeded onto separate compartments and is suitable for keeping the reciprocal paracrine interactions for studying the effect of MSC secretome on tumour migratory, invasive, and stemness potentials (26, 31, 32). Finally, the co-seeding of the two cell populations on the same compartment of the culture support is also possible and has been used, for example, to evaluate the transfer of mitochondria from MSC to breast carcinoma cells (33), or to evaluate the metabolic symbiosis (34). Notably, the co-seeding is the most appropriate 2D approach to resemble the *in vivo* phenotype: it allows the cell-to-cell direct contact interactions. For this model, immunostaining is the easiest way of analysis, combined with the observation of either fixed or live cells through confocal or optical microscopes. However, for more complex molecular analysis, expensive techniques to sort single cells are needed, like immunomagnetic separation or, after the cell transfection with a fluorescent reporter, the cell retrieval by flow cytometry (34, 35). In conclusion, 2D *in vitro* culture systems are easily handled but they are falling short in predicting biological responses since they cannot thoroughly recapitulate both the complexity and the specificity of living tissues. Indeed, tumours are not merely clusters of proliferating cancer cells that grow on plastic 2D surfaces, but rather highly complex 3D structures with a dynamic extracellular matrix (ECM) and reactive stromal cells with a precise spatial relationship. Thus, 2D models should be not used for studying complex processes that cannot be reproduced in this type of culture, like drug perfusion in the tissue, intravasion, or extravasion of tumour cells, invasion of tumour cells through an ECM with a 3D structure, cytotoxicity of anticancer-drugs that might be affected by solute or gas gradient or by different transcriptome or proteome signature that is affected by the tumour-stroma interaction in 3D structures. Based on that, significant effort has been put forward to develop more sophisticated 3D structures, like cells aggregates alone or combined with bioprinting or microfluidics techniques.

The key point to improve 3D co-culturing model is that functional unit of the tissue must be considered rather than single cells, including cell-cell contact and, depending on the cell types, a polarized morphology, a basement membrane and an ECM. For a list and brief description of the most commonly used 3D culturing systems see Table 1. The simplest model of 3D cultures is based on multicellular tumour spheroids without the addition of external ECM component. Despite cells can *per se* secrete ECM proteins, 3D spheroids are commonly considered ECM-free models. Spheroid cultures have been established from several cancers, also to study tumour-MSC interaction, including glioma, breast, colon, ovary, and prostate carcinoma (36). These multicellular structures mimic *in vivo* growth via the formation of a central necrotic core, a solute/ion gradient from the periphery to the centre, and a 3D cellular spatial organization. Forced-floating, hanging drop spheroids, spheroids obtained by using bioreactors are examples of this type of cell aggregates mixed with MSC and tumour cells, at different ratio (e.g., 3:1, respectively) (36, 37). Forced-floating cell aggregates are obtained by avoiding cell attachment to the well bottom. The hanging-drop method is the most widely used and is obtained by seeding a small aliquot of single-cell suspension in a volume that exceeds the well volume. By inverting the plate, the volume generates a drop in which cells are kept in place by surface tension and are then densely packed in spheroid-like structures with high reproducibility. Both forced-floating and hanging-drop spheroids are extensively used for drug screening (38, 39), thanks to the high number of spheroids/plate that can be obtained and the low cost. On the contrary, rotating cell culture bioreactor and spinner flasks force spheroid formation by continuous agitation (37, 40). However, the different size and the fact that spheroids formed in bioreactors must be related to be tested for drug screening, makes them unsuitable for this application. Nevertheless, bioreactors are the best options when long term culture and carefully monitoring of the environmental conditions (such as oxygen and nutrients) are required.

**Table 1:** 3D preclinical models to study the interaction between MSC and cancer cells.

**Model**	**General properties**	**Advantages**	**Disadvantages**	**Applications of the model**
Forced-floating spheroids	Aggregation induced by preventing attachment	Low cost and high reproducibility	Variability in cell size and shape	High-throughput investigations for efficacy vs. toxicity of drugs
Hanging drop	Aggregation induced by agitation at the tip of a drop formed by surface tension	Relative low cost and high reproducibility. Suitable for drug screening and high-throughput testing	The spheroid forms a necrotic core	High-throughput drug screening
Rotating cell culture bioreactors	Forced spheroid formation by continuous agitation	Easy to produce spheroids on a large scale	Specialized equipment required. Variability in size and shape	Ideal for cells that require long-term cultures and controlled amounts of nutrients and oxygen
Scaffold-based	Cells are seeded within a gel-like scaffold of natural or synthetic origin	Provides a 3D support that mimics the physiological tissue for ECM composition	Higher costs. Difficult to retrieve cells from the biomaterial. Lack of reproducibility	3D structures where the cells are free to migrate or form *in vivo*-like cues
3D printing	Cells are printed within scaffold of natural or synthetic origin	Provides a 3D support that mimics the physiological tissue for ECM composition and the spatial organization	Specialized equipment required. Higher costs. Difficult to retrieve cells from the biomaterial. Lack of reproducibility	Allow formation of custom-specific ECMs or scaffolds
Microfluidics	Cells are seeded on microfluidic device that, by using multiple channel and gel-like scaffolds perfused by fluid medium	Provides a 3D support that reconstitute organ-level *in vivo* characteristics. Live observation.	Specialized equipment required. Higher costs. Difficult to retrieve cells from the biomaterial. Lack of reproducibility. N. of cells that can be used is limited	Identification of molecular cellular mechanisms or biomarkers. High-throughput drug screening

To recreate the interstitial space, it is essential to add the ECM component to the multicellular spheroids (41). For this aim, tumour cell and MSC co-cultures can be admixed to high biocompatible scaffolds of natural origin (i.g. collagen, hyaluronan, matrigel, elastin), or synthetic origin (polyethylenglycol, polyvinvyl alcohol, ceramics, or biomaterials), or also ECM isolated from tumour biopsies to mimic microenvironmental conditions (42). Within the scaffold, cells can interact one with the other (essential in the case of MSC-tumour studies), migrate through the pores and re-create *in vivo*-like communication strategies that mimic physiology. More the used matrix resembles the real tumour matrix and more predictive is assumed to be the model. For the addition of ECM in 3D cancer models, 3D bioprinting has stolen the spotlight since it allows the formation of high-resolution 3D structures by dispensing cell-laden biomaterials in a precisely and spatially defined way (43). In this technique, a hydrogel-like pre-polymer solution with encapsulated cells is stored into the ink cartridge that is connected to a printer head. The printer heads are deformed by a thermal or piezoelectric actuators and squeezed to generate bioink droplets of controllable size. However, to date, with very few exceptions (44), the bioprinting has been barely explored to study the MSC and cancer cells interactions.

Finally, tailored innovative platforms that combine spheroid technologies with disease-specific *in silico* models by using microfluidics, the so called “organ-on-a-chip” technologies that reconstitute organ-level *in vivo* characteristics (45, 46), have emerged also in cancer research. Although more expensive and less practical, this cutting-edge approach facilitates the identification of molecular mechanisms behind the disease or the identification of novel biomarkers, and is also particularly useful for drug screening. Microfluidics allows the study of complex phenomena under the combination of multiple biochemical and biophysical parameters, coupled with high-resolution real-time imaging. This type of approach has been mainly developed to study the interaction of tumour cells with blood vessels that can be recreated in the microfluidic chips. Few examples have also been reported for co-culturing MSC with tumour cells, like for lung cancer (47), or to recapitulate the bone metastatic niche that also includes MSC, like for acute lymphoid myeloma, acute lymphoblastic leukaemia, or breast carcinoma (48–50). By using this approach, it is possible to evaluate on real-time the induction of tumour apoptosis, proliferation, migration and invasion, the activation of the reactive stroma, the secretion of cytokines by tumour cells, the activation of specific oncogenes, and stroma-mediated extravasion and intravasion.

## *In vivo* Models

To study the role of MSC on cancer development and progression, several animal models has been developed, mainly xenograft and syngenic small rodents, with MSC co-injected with tumour cells (51–53). In these models, MSC participate to tumour pathophysiology, ultimately facilitating the metastatic spread of weakly metastatic cancer (52, 54–56). MSC/tumour cells ratio seems to be particularly relevant like for tumour dormancy/growth, as demonstrated in melanoma or osteosarcoma models (57, 58). However, the isolation of MSC with different methods and from different tissues (e.g., bone marrow and adipose tissues) have made it difficult to reach consistent conclusions.

Heterotopic injections are the most used and include the subcutaneous injections (52), the easiest and most reproducible model that rarely gives origin to metastases, and that is quite far from the human disease since the host tissue surrounding the tumor might be very different from the tumor-associated stroma of the normal tissue where the tumor develops.

Systemically infused MSC localize within injured, inflamed, and cancerous tissues. Thus, to study the tropism of MSC to the tumour, MSC have been injected into circulation through the tail vein (59, 60). MSC have been also systemically administered via alternative routes, like via intratracheal (61), internal carotid artery (62), intraperitoneal (63), like for gliomas, breast, colon, ovarian, and lung carcinomas (52, 53, 61–66). Systemic injection of MSC may be useful also to enhance their viability along the experiment. Indeed, as it appeared from studies on MSC-based cell therapy, MSC survival is very low (67–70). Thus, periodic injections of MSC after the first injection might enhance their engraftment in the tumour and ensure the continuous secretion of MSC-derived protein factors.

Finally, the use of orthotropic injection site mimics the fate of MSC that have been already chemoattracted by tumour cells and have migrated at the primary tumour site, and it better recapitulates the human disease. However, this model has a great variability and requires a higher number of animals to obtain results that may be correctly interpreted.

It is clear that, due to the short-term survival of injected MSC, monitoring their fate *in vivo* is crucial to support the conclusions about their pro- or anti-cancer activities. As for tumour cells, tracking MSC fate has been obtained by different approaches (see Table 2). In principle, the most powerful technique should allow non-invasive live imaging by optical or not-optical methods to gain real-time information. After animal sacrifice, also histology, immunofluorescence, immunohistochemistry, or flow cytometry techniques on isolated live or fixed cells can be used. Among the live-imaging techniques, the most used are the pre-labelling of MSC with a lipophilic fluorescent dyes (e.g., DiL or Cell TrackerTM) (54), or the pre-tagging of MSC by specific gene transfection, like luciferase or green fluorescent protein for the detection of bioluminescence (60, 71, 84) or fluorescence (55), respectively. Notably, bioluminescent imaging of luciferase-expressing cells is also a quantitative technique for the direct assessment of tumour growth (51, 53), whereas non-optical methods, such as magnetic resonance imaging (MRI) (82), positron emission tomography (PET) (65) and single photon emission computed tomography (SPECT) (83) provide a high spatial resolution and three-dimensional whole-body imaging.

**Table 2:** Methods for imaging MSC-tumour interplay *in vivo*.

**Method**	**Cellular modification**	**Contrast agent**	**Model of implantation**	**Tumour model**	**Reference for tracking MSC**	**Reference for tracking tumour cells**
BLI-live imaging	Luciferase transduction	Bioluminescence from luciferase/luciferin reaction	Orthotopic and heterotopic	Osteosarcoma, breast, ovarian cancers	(53, 59, 60, 63, 71, 72)	(51, 73)
Fluorescent-live imaging	GFP or fluorescent dye labeling of membrane	Fluorescence from fluorescent proteins or fluorescent dyes	Orthotopic and heterotopic	Glioblastoma, gliomas, breast, colon carcinoma	(62, 64, 65, 74–77)	(52, 62, 64, 65)
PET	Genetic modification of cells (PET reporter gene) or uptake of radioisotope labels	Positron-emitting radionucleotides	Heterotopic	Colon cancer, clear cell sarcoma	(65, 72, 78–81)	
MRI	Magnetic nanoparticles added to cells or coupled to ligands	Superparamagnetic iron oxide contrast agent, internalized iron, metal chelates, etc.	Orthotopic and heterotopic	Melanoma, gliomas	(78, 82)	
SPECT	Uptake of radioisotope labels	Radionucleotides (gamma-emitting radioisotopes)	Heterotopic	Breast cancer	(81, 83)	

In conclusion, to develop a more clinical relevant *in vivo* model that accurately reflects the human tumour biology is an urgent need to better predict the response of the tumour to the treatments and for identifying those steps that are crucial for tumour progression to be targeted or prevented for an improved clinical outcome. The addition of MSC in the model is a step forward in this direction, although for the development of *in vivo* pre-clinical models of MSC-tumour cells interaction several parameters need to be carefully considered according to the specific aim, like MSC:tumour cells ratio, the via of co-injection, and the tracking of MSC to check their fate. Last but not least, variables affecting the secretome should be very carefully analysed.

## The Secretome

Studies on MSC-cancer interplay and analyses of cell conditioned media in the mentioned preclinical models have allowed the identification of soluble mediators of the indirect communication between MSC and tumour cells. To summarise, cancer cells frequently secrete IL-1 and TGF-β which switch MSC to a pro-inflammatory phenotype (85, 86), and the monocyte chemotactic protein-1 (MCP-1) which stimulates MSC migration (87). Conversely, MSC produce a plethora of cytokines which, in turn, modulates cancer cell behaviour: IL-6 and IL-8 that promote tumour cell proliferation, survival, migration, and invasion of different tumour cells (26, 88–90), CCL5 that support metastasis in several cancers (52, 91–93), the pro-angiogenic cytokine VEGF that enhances tumour growth and metastasis (94, 95), and TGF-β that fosters tumour invasion and metastasis via EMT (96). Cell communication within the tumour microenvironment is mediated also by exosomes, extracellular nanovesicles that deliver a functional cargo of proteins, lipids, and nucleic acids (97). Tumour-derived exosomes are able to co-opt and reprogram MSC by enhancing their pro-tumourigenic functions, including the pro-angiogenic activity and the production of the pro-inflammatory cytokines IL6 (51, 98). On the other side, exosomes derived from MSC are able to influence tumour development (99), and to increase the tumour stemness (100). Besides, several metabolites, like glutamine, lactate, and ketone bodies, that are released both by tumours cells and by MSC in the extracellular space might fuel the energetic metabolism of cells of the TME (101) or may act as signalling molecules, ultimately stimulating cancer motility, survival, or self-renewal (2, 102–106). Also in osteosarcoma, we recently demonstrated that tumour cells cause an oxidative stress in MSC that, in response, acquire a Warburg phenotype and produce a large amount of lactate.

In this context, it is worth to highlight that the *in vitro* conditions, both 2D and 3D, might induce secretory modifications *per se* (107), thereby affecting the interpretation of results, like by using experimental conditions that can unintentionally exert a stressing stimulus for the cells. Thus, we suggest to evaluate results by considering cells, secretome, and three-dimension as an integrated whole. This add complexity to the system, and careful attention has to be paid when setting up the experiment according to the specific aim and during the interpretation of data.

## Conclusions

Several model systems are now available to characterize the MSC-tumour interplay in the TME. These offer early promise in establishing robust preclinical platforms for the identification of crucial molecular pathways and for the assessment of clinical efficacy of novel drugs to inhibit cancer development and progression. However, selection of the right model for a given study should be shaped on the purpose, and should also consider fixed biological, biochemical, and biophysical parameters according to the specific tumour type. Finally, in order to get reliable and useful results to be translated to the clinic, it should be always kept in mind the careful comparisons in the prediction of human outcomes by the developed model.

## Author Contributions

All authors listed have made a substantial, direct and intellectual contribution to the work, and approved it for publication.

### Conflict of Interest Statement

The authors declare that the research was conducted in the absence of any commercial or financial relationships that could be construed as a potential conflict of interest.
